# lncRNA FER1L4 is dysregulated in osteoarthritis and regulates IL-6 expression in human chondrocyte cells

**DOI:** 10.1038/s41598-021-92474-8

**Published:** 2021-06-22

**Authors:** Jinhai He, Li Wang, Yajun Ding, Hongbing Liu, Guoyou Zou

**Affiliations:** 1 Department of Orthopedics, The Fourth Affiliated Hospital of Nantong University, 166 Yulong West Road, Yancheng, 224005 Jiangsu People’s Republic of China; 2 Department of Orthopedics, The First People’s Hospital of Yancheng, Yancheng, People’s Republic of China; 3 Department of Orthopedics, Yingkou Sixth People’s Hospital, Yingkou, People’s Republic of China

**Keywords:** Diagnostic markers, Genetic markers

## Abstract

Osteoarthritis (OA) is the most prevalent joint disease and is one of the major causes of disability in the world. There has been an increase in the incidence of OA, which is associated with an aging population, sedentary lifestyle, and reduced physical activity. Due to the complex OA pathogenesis, there are limited diagnostic tools. OA is a degenerative joint disorder with a recognized inflammatory component, usually described as abnormal expression of inflammatory factors. For instance, interleukin 6 (IL‐6) has been shown to be upregulated in serum and synovial fluid among patients with OA. Most of the inflammatory factors have been associated with the expression of long noncoding RNAs (lncRNAs). However, the role of the novel lncRNA Fer-1-like protein 4 (FER1L4) in OA is yet to be determined. Here, we interrogated the expression profile of FER1L4 in patients with OA to define its potential application as a diagnostic marker. We collected synovial fluid and blood samples from both OA cases and normal controls. Using qRT-PCR, we evaluated the expression of FER1L4 in plasma and synovial fluid. On the other hand, the expression of IL-6 in plasma and synovial fluid was assessed using ELISA. Besides, the effect of age, gender or disease stage in the expression of the FER1L4 in plasma was also estimated. Moreover, the receiver operating characteristic (ROC) curves were used to determine the impact of FER1L4 in OA cases compared with the normal controls. In addition, we analyzed the correlation between FER1L4 and IL-6 through Pearson correlation analysis. Also, IL-6 expression in overexpressed FER1L4 samples was detected in chondrocytes through western blot analysis, while FER1L4 expression following endogenous IL-6 exposure was detected by qRT-PCR. Our data showed that whereas lncRNA FER1L4 is downregulated in OA patients, IL‐6 is upregulated. The plasma FER1L4 levels among the OA cases were suppressed with disease progression and old age, and the down-regulation could efficiently discriminate OA patients from normal subjects. In addition, upregulation of FER1L4 inhibited IL‐6 expression in human chondrocyte cells, and treatment with different concentrations of exogenous IL‐6 did not affect the expression of FER1L4. Taken together, our data demonstrates that FER1L4 could efficiently identify OA cases from normal subjects, and can also modulate the expression of IL‐6 in human chondrocytes.

## Introduction

Osteoarthritis (OA) is the most prevalent joint disease, which mostly affects the spine, hips, knees or fingers. OA leads into joint pain, synovitis, progressive degradation of cartilage, as well as changes in subchondral bone and periarticular tissues^1,2^. Typically, pain is the frequently reported initial clinical manifestation of OA, which commonly results in disability^3,4^. Risk factors such as aging, obesity and elevate the incidence of OA^5^. Whereas there are pharmacological or non-pharmacological treatment options for OA, they only improve the symptoms and not structural changes^6–8^. Besides, many animal model studies have tried to develop novel tools in the management of OA, but few have succeeded in their clinical efficacy. Thus, it is important to increase investigations towards the pathogenesis of OA^9–11^. The OA pathogenesis often triggers inflammatory responses. OA was recognized as inflammation-related disease, whose development and deterioration are frequently related to the abnormal expression of inflammatory factors^2,12^. For example, Interleukin 6 (IL‐6) is upregulated in patients with OA^13–16^.

Previous studies have shown that lncRNAs play vital roles in the pathogenesis and progression OA^17,18^. For instance, lncRNA TMSB4 promotes cartilage degradation in OA by sponging miRNA-152^19^. On the other hand, HOTAIR facilitates OA progression by regulating FUT2 expression as well as Wnt/β-catenin pathway^20^. Recognized as a pro-osteogenic lncRNA, fer-1 like family member 4 (FER1L4) expression shows gradual upregulation during osteogenic induction of human periodontal ligament stromal cells (PDLSCs)^21^. In addition, overexpression of FER1L4 promoted osteogenic differentiation of PDLSCs while its downregulation inhibits the differentiation^21^. Besides, other studies demonstrated that FER1L4 promotes VEGFA expression through sequestration of miR-874-3p. VEGFA has been shown to participate in OA^22,23^. Targeting VEGF or its receptors to inhibit VEGF signaling showed could be a potential stand-alone or adjuvant therapy for OA^24^. Since FER1L4 regulates the VEGFA expression, we hypothesized that FER1L4 might be involved in the OA pathogenesis and progression. However, the biological role and mechanisms of FER1L4 in OA development remain unknown.

In our study, we investigated the role of FER1L4 and its correlation with IL-6 in OA cases. We found that FER1L4 is significantly decreased in plasma and synovial fluid in OA patients, and was associated with OA progression and age. On the contrary, levels of IL-6 in plasma and synovial fluid in OA patients were remarkably increased, which exhibited a negative correlation between FER1L4 and IL-6 in OA cases but not in healthy controls. Furthermore, we showed that over-expression of FER1L4 could suppress IL-6 expression, but treatment of IL-6 did not alter FER1L4, suggesting that FER1L4 was an up-stream regulator of IL-6 in OA progression. Thus, our findings indicated that FER1L4 played an important role in OA progression n and may serve as a biomarker for OA diagnosis.

## Materials and methods

All the participants or their legal guardians gave written informed consent. The study protocols were approved by the Ethics Committee of the First Affiliated Hospital of Harbin Medical University and were in accordance with the Declaration of Helsinki.

### Blood samples, synovial fluid, and human chondrocyte cell lines

We collected 81 or 49 blood samples from clinical OA cases or 49 healthy controls, respectively. The patients were admitted at the First Affiliated Hospital of Harbin Medical University between January 2016 and January 2018. We included patients diagnosed with knee OA. Patients who received treatment 3 months prior to commencement of the study, those with additional conditions, as well as those with additional inflammatory disorders, such as colds and pharyngitis were excluded. Besides, patients with other forms of arthritis were also excluded. The collected blood specimens centrifuged for 15 min at 1200 g in EDTA tubes under ambient temperature. We collected plasma for further analysis. On the other hand, synovial fluid was obtained from knee joints of 19 OA cases and 16 healthy controls. The blood or synovial fluid was collected at 8 o'clock in the morning of the same day on an empty stomach. Since the amount of synovial fluid in the knee joint is limited in normal people, all the volunteers participating in the synovial fluid collection were examined by Magnetic Resonance Imaging (MRI) before collection. For the MRI, we used the coronal T2 microscope which could show the synovial fluid. Participants whose knee synovial fluid was less than 2 ml were excluded, and 0.5 ml of synovial fluid was collected from each participant for the assays. There were 40 male and 41 female patients, aged between 34 and 69 (mean, 49.7 ± 6.3) years. On the other hand, there were 24 male and 25 female healthy subjects, aged between 32 and 68 (mean, 49.4 ± 5.6) years. No significant differences were observed in age distribution or gender between the two groups.

Human chondrocytes (Sigma-Aldrich) were cultured in DMED media containing 10% fetal bovine serum (FBS) under 37 °C and 5% CO_2_.

### Cell transfection

FER1L4 over-expression plasmid (FER1L4) and negative control plasmid (NC) were synthesized by Sangon (China). The human chondrocytes were transiently transfected using Lipofectamine 2000 (Invitrogen Carlsbad, USA) following the manufacturer’s protocol. Briefly, the human chondrocytes were seed into 6 cm dishes before transfection. 24 h later, the cells were transfected with control plasmid or FER1L4 over-expression plasmid using Lipofectamine 2000 in serum-free medium. After 5 h, the serum-free medium was replaced with complete medium. After 48 h, the cells were harvested and lysed for further experiments.

### Western blotting assay

Radioimmunoprecipitation assay solution (Thermo Fisher Scientific) was used to extract total proteins from the cells. The proteins were resolved in 10% SDS-PAGE, and then transferred onto PVDF membranes. The blots were blocked with 5% skimmed milk for 1 h, and then incubated with primary antibodies; β-actin (Abcam, Cat#: ab8227, 1:4000) or IL‐6 (Abcam, Cat#: ab229381, 1:1600) for 1 h at room temperature. Thereafter, the blots were incubated with a secondary antibody (1:1000, Beyotime, China) for another 1 h and then developed using ECL (Sigma‐Aldrich).

### Real‐time quantitative PCR (qRT-PCR)

The Trizol reagent (Thermo Fisher Scientific) was used to extract total RNA in plasma and synovial fluid following the manufacturer’s protocols. SuperScript III Reverse Transcriptase (Thermo Fisher Scientific) was used to construct cDNA through while SYBR Green Quantitative RT‐qPCR Kit (Roche) was employed for PCR, following the specified protocols. The primer sequences used for the PCR were as follow:

FER1L4 Forward: 5'-TCACAGACATGGGTGGCAATG -3’,

FER1L4 Reverse: 5'- GGGTTCACAAACCAGTTGAAGG -3';

β-actin Forward: 5'-TTCCAGCCTTCCTTCCTGGG-3',

β-actin Reverse: 5'-TTGCGCTCAGGAGGAGCAAT-3'.

### Enzyme‐linked immunosorbent assay (ELISA)

Plasma IL‐6 levels were detected by the human ELISA Kit (Thermo Fisher Scientific) in accordance with the specified protocols.

### Statistical methods

Differences between the groups were analyzed by student’s t-test. On the other hand, differences in more than 2 groups were compared by one- or two-way ANOVA. In addition, the receiver operating characteristic (ROC) curves were used to determine the significance of FER1L4 in diagnosing OA, where the true positive patients represented OA cases while the true negative ones were regarded as normal controls. Besides, Pearson’s correlation analysis was used to analyze the association between FER1L4 expression and IL‐6. A *P < 0.05 was considered as statistically significant.

### Ethics approval

This study has passed the review of the Ethics Committee of the First Affiliated Hospital of Harbin Medical University.

### Informed consent

All patients signed the informed consent forms.

### Consent for Publication

All authors agree with the content of the manuscript.

## Results

### IL-6 and FER1L4 showed abnormal expression in OA patients.

**Figure 1:**
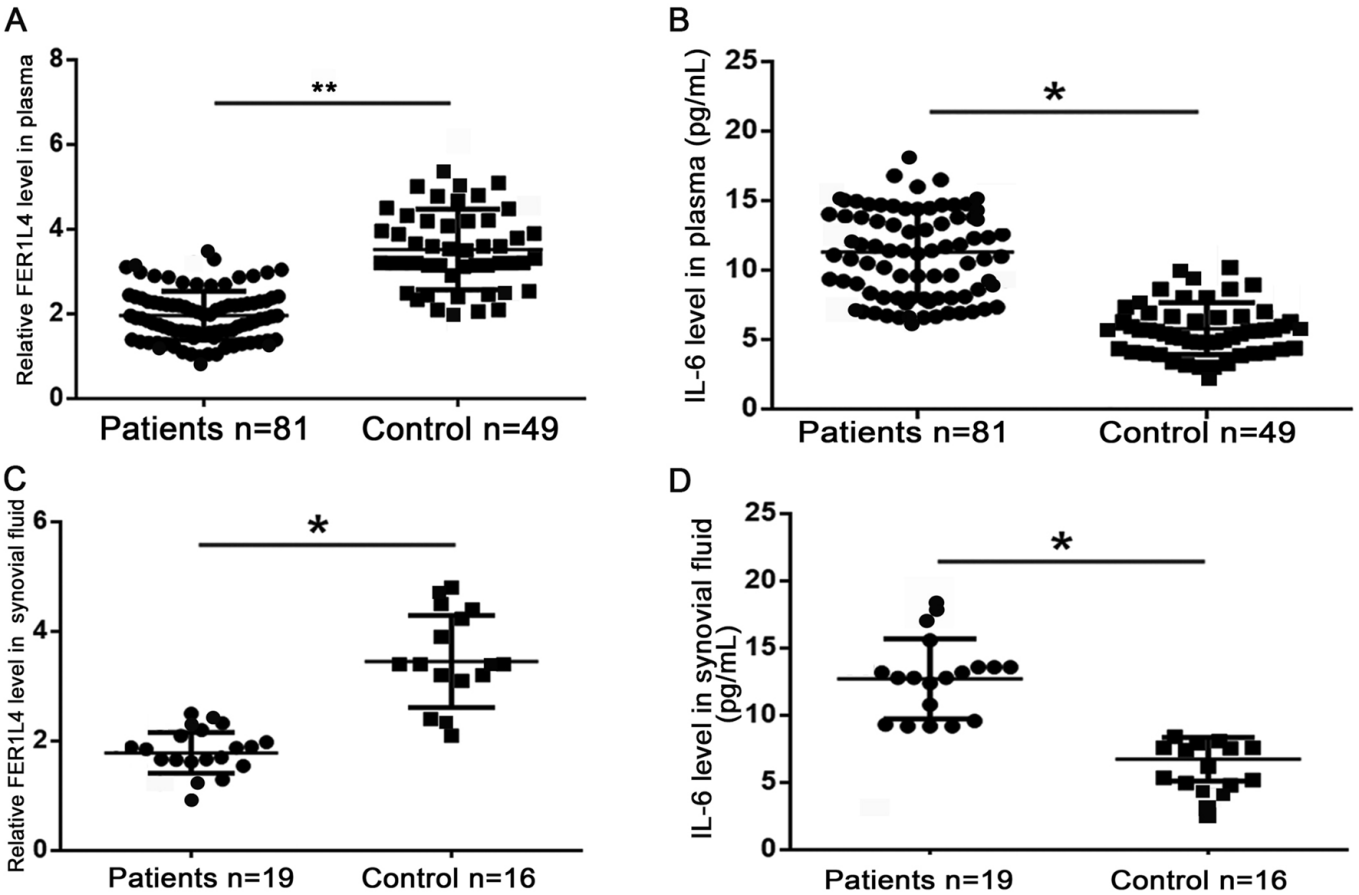
lncRNA FER1L4 and IL‐6 were dysregulated in OA patients. (**A**) qRT‐PCR data showing the FER1L4 expression in plasma of OA patients and healthy controls. (**B**) ELISA results showing the IL-6 expression in plasma of OA patients and healthy controls. (**C**) qRT‐PCR data showing the expression of FER1L4 in synovial fluid of OA patients and healthy controls. (**D**) ELISA results showing the expression of IL-6 in synovial fluid of OA patients and healthy controls.

To evaluate the expression profile of FER1L4 in OA, we performed qRT-PCR using plasma samples from both the patients and healthy controls. The data showed downregulation of plasma FER1L4 among the OA cases relative to the normal subjects (Fig. 1A). On the other hand, ELISA results showed that plasma IL-6 was upregulated in OA patients compared with that in the control group (Fig. 1B). In addition, qRT-PCR for FER1L4 expression in synovial fluid samples showed decreased expression in OA patients compared with that in controls (Fig. 1C). Besides, ELISA assays showed increased IL‐6 expression in synovial fluid from the OA patients compared with that in the control group (Fig. 1D).

### The expression of FER1L4 in OA patients decreased with aging

For investigate the effects of age on the FER1L4 expression, we categorized the OA cases and normal subjects into 3 groups based on age: 40–50, 50–60 and 60–70 years old. Our analysis showed that a markedly decreased plasma FER1L4 expression in the 50–60 years old group compared to the 40–50 years old group. In addition, there was a downregulation of FER1L4 expression in the 60–70 years old group relative to 50–60 years old group (Table 1). On the other hand, there were no obvious changes detected among the three groups in plasma of the normal subjects (Table 1). Similarly, the FER1L4 expression in synovial fluid was significantly decreased in elderly OA patients, with the lowest FER1L4 expression observed in the 60–70 years old group (Table 2).

**Table 1:** FER1L4 was decreased with age in plasma of OA patients not in healthy control. (A) Relative expression of FER1L4 in different age groups of OA patients. (B) Relative expression of FER1L4 in different age groups of healthy controls.

Age	Relative expression of FER1L4	t	*P*
**(A)**			
40–50	3.73 ± 0.56		
50–60	2.74 ± 0.41*	3.327	< 0.05
60–70	1.03 ± 0.12**	7.251	< 0.05
**(B)**			
40–50	3.92 ± 0.48		
50–60	3.12 ± 0.71	1.715	0.235
60–70	4.12 ± 0.89	2.154	0.078

*P < 0.05; **P < 0.01.

**Table 2:** FER1L4 was decreased with age in synovial fluid of OA patients not in healthy control. (A) Relative expression of FER1L4 in different age groups of OA patients. (B) Relative expression of FER1L4 in different age groups of healthy controls.

Age	Relative expression of FER1L4	t	*P*
(**A**)			
40–50	3.87 ± 0.42		
50–60	2.96 ± 0.32**	3.175	< 0.05
60–70	1.84 ± 0.17**	6.725	< 0.05
(**B**)			
40–50	4.02 ± 0.51		
50–60	3.96 ± 0.61	1.182	0.477
60–70	3.82 ± 0.76	1.781	0.324

**P < 0.01.

### The expression of FER1L4 is downregulated with OA progression.

To evaluate the FER1L4 expression shifts with OA development, we classified the OA cases into stage III and IV, based on the Kellgren-Lawrence clinical diagnosis. Then, qRT-PCR was conducted to detect plasma FER1L4 levels in the two groups. The data showed that, compared to stage III, plasma FER1L4 expression was markedly decreased in stage IV (Table 3). Similarly, FER1L4 expression in synovial fluid was significantly decreased in stage IV relative to that in stage III (Table 4).

**Table 3:** Relative expression of FER1L4 in plasma of different stages of OA patients.

Stage	Relative expression of FER1L4	t	*P*
Stage III	2.15 ± 0.51		
Stage IV	0.86 ± 0.68*	2.486	< 0.05

**P < 0.01.

**Table 4:** Relative expression of FER1L4 in synovial fluid from different stages of OA patients.

Stage	Relative expression of FER1L4	t	*P*
stage III	3.24 ± 0.71		
Stage IV	1.28 ± 0.53*	3.814	< 0.05

### FER1L4 downregulation distinguished OA patients from the healthy controls.

**Figure 2:**
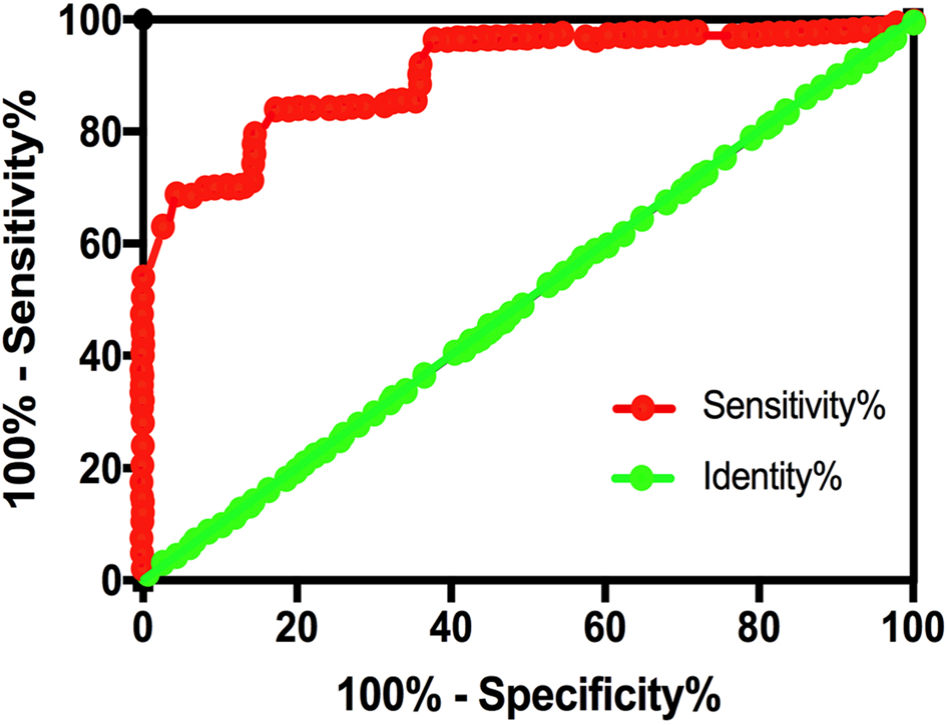
lncRNA FER1L4 downregulation could effectively distinguish OA patients from healthy controls. The diagnostic values of plasma FER1L4 was analyzed using ROC curve.

To interrogate the diagnostic ability of the FER1L4 expression profile, we analyzed the efficiency in classifying the uniformly distributed samples by ROC curves. In the ROC curves, the false positive rates (FPRs) were used as X-coordinates, while the true positive rates (TPRs) were used as Y -coordinates. Besides, the real positive cases indicated the OA cases, while the negative ones indicated normal subjects. Furthermore, an AUC value of 0.5–1 indicated that the model outperformed random guessing. Predictive value was obtained when the appropriate thresholds were set by the model. Our data showed that the AUC value was 0.9221, with a standard error of 0.02088%, and a 95% confidence interval (CI) was 0.8909–0.9732 (Fig. 2). Such true positive result indicated that the FER1L4 expression could efficiently distinguish the OA cases from the healthy subjects.

### No influence of gender on the levels of FER1L4 and IL-6

Studies have showed that higher incidence of OA was observed in females than in males. Thus, the relationships of levels of FER1L4 and IL-6 with gender among the OA cases were examined. OA cases were divided into male or female group for analysis. Unexpected, no significant change was observed in FER1L4 expression between the male and female OA cases (Table 5). Similar results were obtained among normal subjects, which suggested no influence of gender on the FER1L4 level (Table 5). Similarly, the level of IL-6 was not affected by gender (Table 6).

**Table 5:** No difference of FER1L4 expression was detected between in male or female specimens. (A) Relative levels of FER1L4 expression in male or female OA patients. (B) Relative levels of FER1L4 expression in male or female healthy controls.

Gender	Relative expression of FER1L4	t	*P*
(**A**)			
Male	2.12 ± 0.45		
Female	2.23 ± 0.41	1.425	0.536
(**B**)			
Male	3.73 ± 0.42		
Female	3.58 ± 0.63	1.159	0.098

*P < 0.05.

**Table 6:** No difference of IL-6 expression was detected between in male or female specimens. (A) Relative levels of FER1L4 expression in male or female OA patients. (B) Relative levels of FER1L4 expression in male or female healthy controls.

Gender	Level of IL-6 (pg/ml)	t	*P*
(**A**)			
Male	13.34 ± 4.11		
Female	12.86 ± 5.92	5.751	> 0.05
(**B**)			
Male	5.78 ± 2.25		
Female	6.56 ± 1.87	4.278	> 0.05

*P < 0.05.

### IL‐6 is negatively correlated with FER1L4 in OA patients

**Figure 3:**
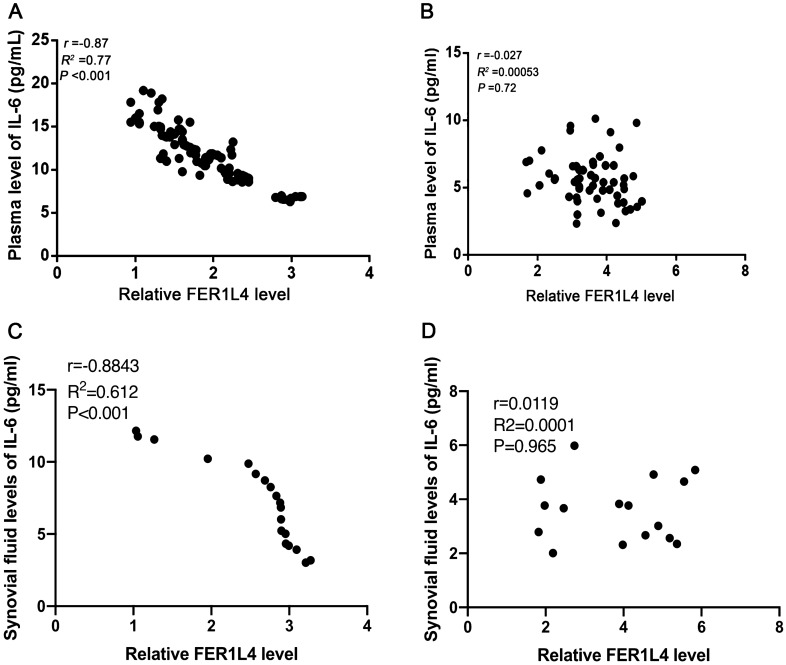
FER1L4 and IL‐6 were inversely correlated in OA patients Pearson's correlation coefficient analysis was performed to analyze the correlation between of FER1L4 and IL-6 in plasma of OA patients (**A**) and healthy controls (**B**), as well as in synovial fluid of OA patients (**C**) and healthy controls (**D**).

To evaluate the relationship between FER1L4 and IL-6, we determined the association of plasma FER1L4 expression with IL-6 among the OA cases or healthy controls through Pearson correlation analysis. The data showed that FER1L4 was negatively correlated with IL‐6 in the patients with OA (r = − 0.87, *p* < 0.001) (Fig. 3A), but not among healthy controls (r = − 0.027, *p* = 0.72) (Fig. 3B). Similarly, FER1L4 expression in the synovial fluid was negatively correlated with IL-6 levels among OA cases (Fig. 3C), but not in the healthy controls (Fig. 3D).

### FER1L4 suppressed IL‐6 expression in human chondrocytes

**Figure 4:**
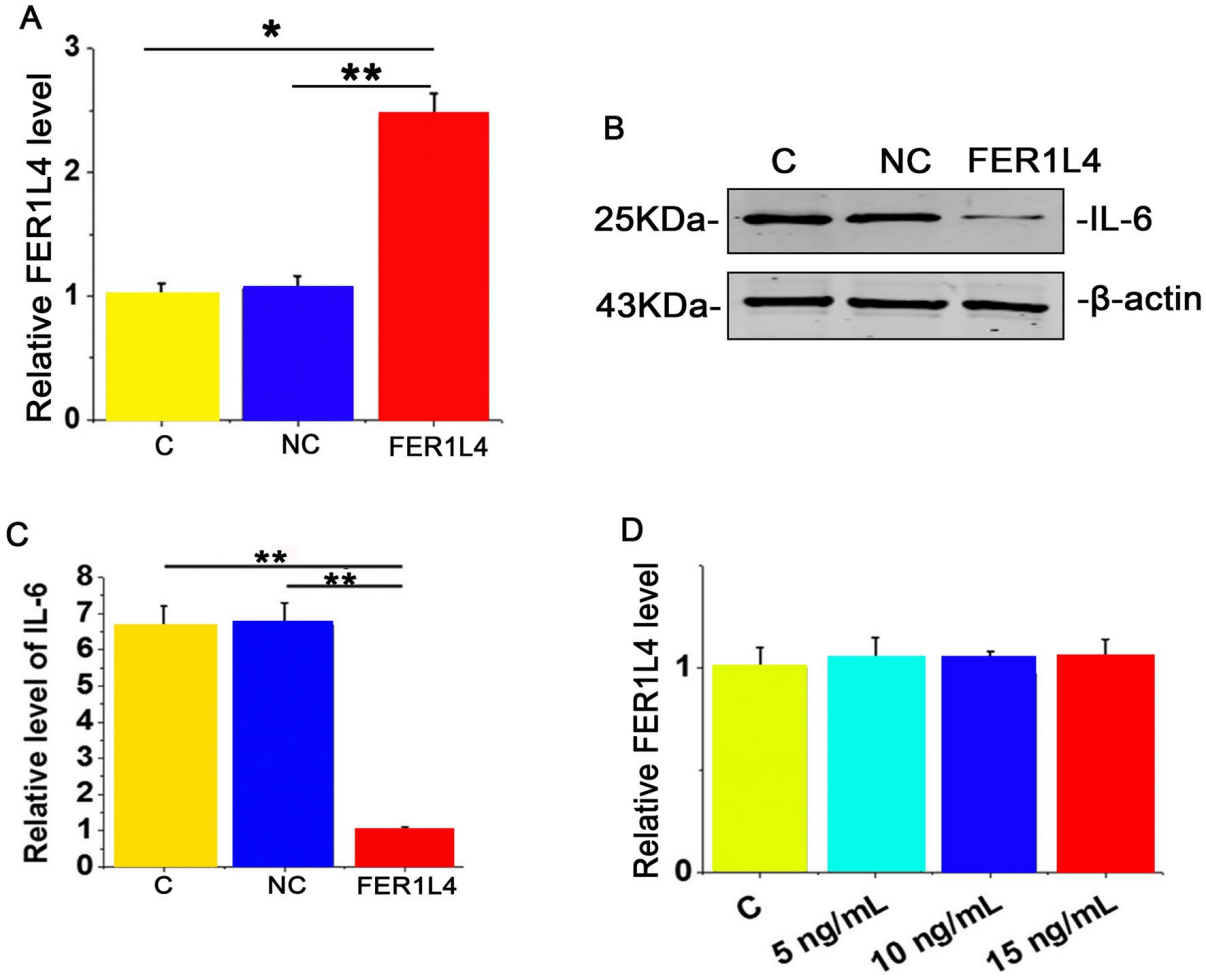
FER1L4 inhibited IL‐6 expression in human chondrocyte cells. (**A**) qRT‐PCR results showing the relative expression of FER1L4 in human chondrocyte cells after FER1L4 or NC transfection. (**B–C**) Western blot results showing the expression of IL-6 in human chondrocyte cells after FER1L4 or NC transfection. (**D**) qRT‐PCR results showing the level of FER1L4 expression in human chondrocyte cells after treatment with IL-6. Data are represented as means ± SEM of 3 independent experiments. *P < 0.05.

To analyze the effect of FER1L4 expression on IL-6 in chondrocytes, the FER1L4 over-expression plasmid (FER1L4) or the negative control plasmid (NC) was transfected into human chondrocytes (Fig. 4A). Compared to the NC group, FER1L4 overexpression decreased the expression of IL-6 protein within human chondrocytes (Fig. 4B-C). Besides, human chondrocytes were exposed to exogenous IL-6 treatments at 5, 10 and 20 ng, and then FER1L4 levels were detected by qRT-PCR. The data showed that exogenous treatment with IL-6 protein did not affect the expression of the FER1L4 in these cells (Fig. 4D). Thus, FER1L4 regulates IL-6 expression in human chondrocytes.

## Discussion

OA, a musculoskeletal disorder reduces patient mobility, quality of life and productivity, while increasing the economic burden in patients and society^25–27^. OA affects about 240 million people globally and is predicted to become a musculoskeletal disorder with the highest prevalence by 2040^28,29^. Besides, OA has been shown to elicit inflammatory reactions^30^. Two typical inflammatory molecules, IL-6 and tumor necrosis factor-a (TNF-a) are implicated in OA progression^31^. Although both IL-6 and TNF-a are increased in OA samples, over-expression of FER1L4 only suppressed the expression of IL-6 but not TNF-a (Fig. S1). Our findings suggested that the upregulated IL-6 in OA is as a result of suppressed FER1L4 expression.

FER1L4 is a newly discovered lncRNA and is dysfunctional in cancers such as liver cancer, colon cancer and endometrial cancer^32,33^. Previous studies have shown that FER1L4 plays anti-tumor roles in suppressing cancer cell proliferation and metastasis as well as inducing cancer cell apoptosis^32,34,35^. Besides, FER1L4 plays oncogene-like role in glioma cells^36^. In addition, FER1L4 regulates rheumatoid arthritis by modulating NLRC5^37^. Overexpression of FER1L4 has been shown to decrease the expression of NLRC5 as well as inflammatory cytokines, thus playing an anti-inflammation role^37^. Although the FER1L4 expression in male and female OA patients is similar, we showed suppression of FER1L4 expression among OA cases, and the decline was associated with age and stage of OA. These observations demonstrated that FER1L4 might serve as the biomarker in OA diagnosis.

On the other hand, IL-6 is a well-studied and potent factor in numerous inflammatory diseases^38–41^. It has been shown that IL‐6 suppression can be used to manage inflammatory diseases^42–44^. Besides, IL-6 is aberrantly over-expressed in OA^45^. Higher expression of IL-6 is associated with severe OA status^14^. In our study, we showed that FER1L4 expression was negatively correlated with IL-6 in OA patients. In addition, whereas over-expression of FER1L4 resulted in reduced levels of IL-6, IL-6 treatment could not affect FER1L4 expression, thus FER1L4 may act as an upstream regulator of IL-6. However, such regulatory mechanisms were not detected in normal subjects. These data suggested an indirect regulation of FER1L4 on IL-6, which affects the progression of OA.

## Conclusion

Taken together, our data showed that FER1L4 could be an effective biomarker for the detection of OA and regulates the IL‐6 expression in human chondrocytes.

## Supplementary Information

Supplementary Information.

## Abbreviations

lncRNA: Long non-coding RNA
OA: Osteoarthritis
IL‐6: Interleukin 6
NC: Negative control plasmid
qRT-PCR: Real‐time quantitative polymerase chain reaction
ELISA: Enzyme‐linked immunosorbent assay
ROC: Receiver operating curve
