# Supplementary material for: lncRNA FER1L4 is dysregulated in osteoarthritis and regulates IL-6 expression in human chondrocyte cells
Source: Sci Rep. 2021 Jun 22;11:13032. doi: 10.1038/s41598-021-92474-8 (PMC8219729; doi:10.1038/s41598-021-92474-8)

**lncRNA FER1L4 is dysregulated in osteoarthritis and regulates IL-6 expression in human chondrocyte cells**

**Figure S1**


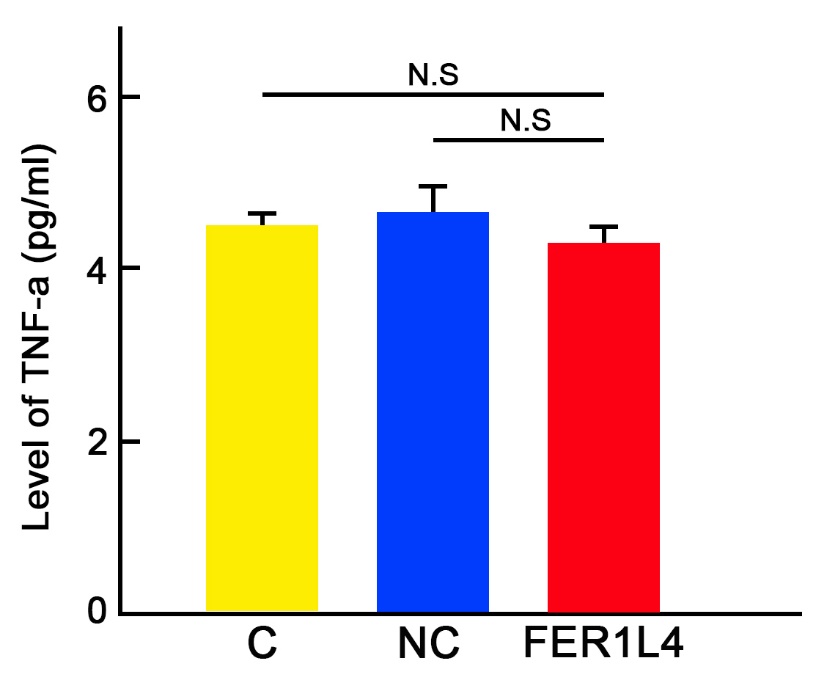


**Figure S1: Over-expression of FER1L4 did not altered TNF-a expression.** Human chondrocyte cells were transfected with FER1L4 expression vector or negative control plasmid, respectively. After 48 hours transfection, the supernatants were collected and the levels of TNF-a in supernatants were examined by ELISA.

**Unedited blots**

**Figure 4B**


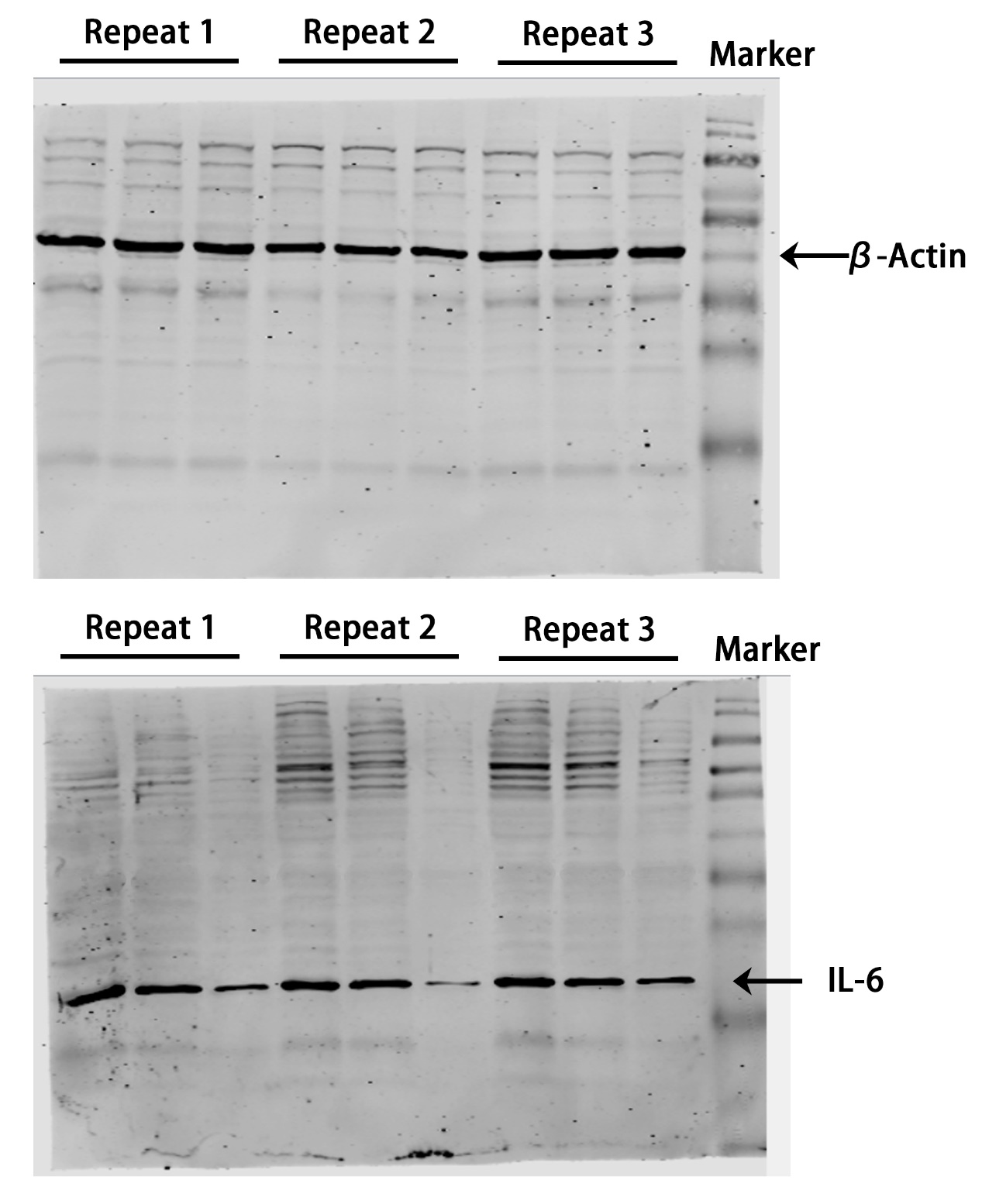

Supplement: Supplementary file 1 — Supplementary Information. [file 41598_2021_92474_MOESM1_ESM.docx]
